# Deciphering the Role of Mast Cells in HPV-Related Cancers

**DOI:** 10.3390/ijms262412110

**Published:** 2025-12-16

**Authors:** Zyanya P. Espinosa-Riquer, J. Omar Muñoz-Bello, Claudia González-Espinosa, Alfredo Ibarra-Sánchez, Marcela Lizano

**Affiliations:** 1 Unidad de Investigación Biomédica en Cáncer, Instituto Nacional de Cancerología, Mexico City 14080, Mexico; espinosariquer@gmail.com (Z.P.E.-R.); jmunozb@incan.edu.mx (J.O.M.-B.); 2 Departamento de Farmacobiología y Centro de Investigación sobre el Envejecimiento, Centro de Investigación y de Estudios Avanzados, Unidad Sede Sur, Mexico City 14330, Mexico; cgonzal@cinvestav.mx (C.G.-E.); aibarra@cinvestav.mx (A.I.-S.); 3 Departamento de Medicina Genómica y Toxicología Ambiental, Instituto de Investigaciones Biomédicas, Universidad Nacional Autónoma de Mexico, Ciudad Universitaria, Mexico City 04510, Mexico

**Keywords:** HPV, mast cells, oncogenes, oncoproteins, cancer, cervical cancer, HNSCC, pro-tumoral, anti-tumoral

## Abstract

Human Papillomavirus (HPV)-related cancers constitute a major global health problem, accounting for 4.5% of all human cancers. Studying the composition of the tumor microenvironment (TME) of HPV-related cancers may help develop therapeutic strategies or identify prognostic biomarkers with potential clinical significance. Among all the components of TME, mast cells (MCs) appear to be particularly relevant in HPV-related tumors. MCs are myeloid-derived immune cells that release a wide range of inflammatory mediators. It is now recognized that these immune cells are important players within the TME, where they can exert both anti- and pro-tumor activities depending on the type of MC-derived inflammatory mediators released. MCs may play an important role in the processes associated with cell transformation, development, and the progression of HPV-associated tumors; however, their specific functions in these neoplasms are not yet fully understood. This review addresses the current state of knowledge on MCs and their contribution to the molecular biology of HPV-related cancers. In addition, it highlights MCs’ roles in the pro- or anti-tumor paradigm and discusses their emerging potential as therapeutic targets or prognostic biomarkers.

## 1. Introduction

Human Papillomavirus (HPV) is estimated to be responsible for approximately 640,000 new cases of cancer each year worldwide and accounts for about 30% of cancer cases associated with infectious biological agents [1]. Due to their association with cancer, HPVs are classified as low-risk (LR-HPV) and high-risk (HR-HPV) of which HR-HPVs are considered etiological factors of several cancer types, with HPV16 and HPV18 being the most frequently found in HPV-associated tumors [2]. Cervical cancer (CC) is the type of cancer most commonly associated with HPV infection, and virtually all cases are HPV-positive, while HPV is found in 88% of anal cancer cases, and in 50%, 78% and 25% of penile, vaginal, and vulvar cancers, respectively [3]. Additionally, about 25% of head and neck cancer (HNC) cases are HPV-positive [4], and the cumulative HPV burden in oropharyngeal cancer is reported to be 33% worldwide [5]. Epidemiological data reveal that, despite prophylactic and early detection strategies, the number of tumors associated with HPV infections continues to rise, particularly in oropharyngeal and anal/rectal cancers [6].

The interaction between cancer cells and their microenvironment can lead to both tumor progression and tumor suppression. Tumors can recruit other cell types to their environment and form the tumor microenvironment (TME), which is composed of cellular and non-cellular elements. In addition to cancer cells, the TME includes cellular components such as immune, stromal, and endothelial cells, forming blood and lymphatic vessels, fibroblasts, and adipocytes, while non-cellular components include the extracellular matrix, proteolytic enzymes such as matrix metalloproteinases, and growth factors, among others [7]. Tumor infiltrating immune cells include neutrophils, macrophages, mast cells (MCs), myeloid-derived suppressor cells, dendritic cells, natural killer cells, and T and B lymphocytes [7,8].

The TME in HPV-positive tumors differs substantially from that of HPV-negative tumors, especially in HNC. Traditionally, HPV-positive tumors have been considered immunologically “hot”, also referred to as infiltrated/inflamed, and are characterized by increased infiltration of immune cells, elevated levels of pro-inflammatory mediators, lower levels of COX2, and increased PD-1 expression when compared to HPV-negative tumors [9,10]. In contrast, HPV-negative tumors have been described as immunologically “cold” or infiltrated/excluded, and are defined by the absence of cytotoxic T lymphocytes and pro-inflammatory mediators within the tumor core [10]. These data indicate a clear difference in the immunological landscape of HNC with respect to HPV infection. However, recent evidence shows that HPV-positive tumors are far more heterogeneous than previously thought. Transcriptomic studies in HNC samples have demonstrated that not all HPV-positive tumors present a uniformly “hot” phenotype. Instead, HPV-positive HNCs can be classified into three different immune subgroups: immune rich, mixed, and immune desert. The immune-rich group has the classical characteristics of HPV-positive tumors, with abundant lymphocyte infiltration and strong immune activation, and is associated with the most favorable clinical outcomes. In contrast, the immune-desert group shows minimal immune infiltration despite being HPV-positive, resembling HPV-negative tumors and exhibiting worse survival rates [11]. Therefore, in HPV-positive HNC tumors, the degree of intra-tumoral immune-cell infiltration is strongly associated with an improved response to standard therapy and with a favorable clinical outcome. It is worth mentioning that MCs are not represented in these studies; however, their participation in the establishment of “hot” and “cold” tumors cannot be ruled out.

MCs are immune cells derived from the myeloid lineage that reside in tissues and contain granules loaded with potent inflammatory mediators such as histamine. MCs have been identified in several types of tumors, where they can exert either pro- or anti-tumor activity. These tumor-infiltrating cells may originate from resident sentinel cells or from recruited progenitor cells, and they can directly influence cancer cell proliferation, migration, and invasion, or indirectly shape the TME and modulate immune responses to tumor cells [12].

MCs express a wide range of surface receptors that can be activated in response to microenvironmental stimuli, leading to the secretion of several molecules, including growth and pro-angiogenic factors, an well as pro- and anti-inflammatory mediators that influence tumor growth [13]. Several studies have attempted to elucidate the involvement of MCs in tumor progression and patient clinical outcomes. Given the conflicting evidence across tumor types, both the pro-tumoral and anti-tumoral functions of MCs have been proposed. For instance, the presence of MCs in the stroma of invasive breast carcinoma tumors correlated with a good prognosis [14,15]. In contrast, a higher MC density has been observed in poor-grade and high TNM stage colorectal tumors [16]. Furthermore, high tumor infiltration by MCs in CC has been associated with worse overall survival [17].

This review focuses on the paradigms related to the pro- and anti-tumor functions of MCs in HPV-related tumors. Here, we discuss the contribution of MCs to the establishment of the TME and the progression of HPV-positive cancers. In addition, we examine the association between MC density and clinical outcomes in patients with HPV-positive tumors, as well as the potential therapeutic implication of targeting MC-derived inflammatory components.

## 2. Human Papillomavirus (HPV) Infection and Cancer Establishment

HPVs are non-enveloped small viruses, containing a circular double-stranded DNA genome of 8000 bp that infects mucosal and cutaneous epithelia [18]. The HPV genome is divided into three regions: the long control region (LCR), which contains regulatory sites for viral replication and transcription; the early region which includes the E6, E7, E1, E2, E4, and E5 genes whose protein products regulate the viral replication cycle; and the late region which contains L1 and L2 genes that encode the viral capsid proteins. E6 and E7 are the main viral oncoproteins that induce delayed differentiation, viral replication, and the evasion of host immunity [19,20]. HPV infects basal epithelial cells through microlesions, where it remains in a low copy number (approximately 50–100 episomal copies per cell). As epithelial cells divide and differentiate, the productive phase of the viral cycle begins, resulting in massive viral genome amplification and the transcription of late genes, including L1 and L2 for virion assembly. Finally, viral particles are released from the differentiated upper epithelial layers, allowing the infection of new cells. In fewer than 10% of cases, a persistent infection is established, a condition that may last for years and significantly increases the risk of cancer development [21].

The oncogenic role of HPV was elucidated 40 years ago and was first described in CC. Approximately 1% of cervical HPV infections progress to cancer [22]. During a persistent infection, the HPV genome frequently integrates randomly into the host genome, often breaking within the E1 and E2 genes, which results in loss of their expression. This event leads to the overexpression of the E6 and E7 oncogenes, since their transcription was previously repressed by the E2 protein [23].

Following viral DNA integration, the overexpressed E6 and E7 oncoproteins increase their interaction with a plethora of cellular proteins that regulate the cell cycle, metabolism, apoptosis, and immune response. Extensive research has been conducted to elucidate how HPV oncoproteins contribute to cancer. The continuous expression of E6 and E7 promotes cellular transformation by deregulating processes such as proliferation, differentiation, immune evasion, apoptosis, immortalization, and genome instability, among other cancer-associated pathways [24]. One of the best-described activities of the E6 protein is the formation of a complex with p53 and the E6AP ubiquitin ligase, which promotes ubiquitin-dependent proteasomal degradation of a major fraction of the p53 pool [25]. This abrogates the transcription of p53-responsive genes and disrupts cell cycle arrest and apoptosis in response to cellular stress. E6 also promotes cellular immortalization by inhibiting senescence, largely through the degradation of the telomerase inhibitor NFX1-91 and with the participation of c-Myc; the hTERT catalytic subunit is then efficiently transactivated, increasing telomerase activity [26].

The main cellular target of the E7 oncoprotein is the retinoblastoma tumor suppressor protein (pRb), which negatively regulates the transcription factor E2F when bound to it. E7 forms a complex with the ubiquitin ligase cullin-2, inducing the degradation of pRb and the subsequent release of E2F. This enables the expression of E2F-dependent genes, such as cyclins A and E, which promote cell cycle progression [27]. E7 also promotes the degradation of p107 and p130, which repress E2F function [28,29]. Furthermore, E7 stimulates cell proliferation by inhibiting the cyclin-dependent kinase (CDK) inhibitors p21WAF1/CIP1 and p27KIP1 [30,31].

Another actor in the HPV transformation process is the E5 oncoprotein, whose functions are not completely elucidated. E5 is a hydrophobic low-molecular weight protein localized mainly in the plasma and nuclear membranes, endoplasmic reticulum, and Golgi apparatus [32]. Although E5 alone exhibits weak transforming activity in vitro, its transforming effects appear to be mainly mediated through the epidermal growth factor receptor (EGFR) signaling pathway [33].

HPV has a prolonged replication cycle and can establish long-term persistent infections, which require the action of viral mechanisms to evade host immune response [34]. The E5, E6 and E7 oncoproteins help circumvent host immune defenses, promoting the establishment of persistent infections that may ultimately drive cancer development. E5 contributes to immune response evasion by interacting with major histocompatibility complex class I (MHC-I) molecules, retaining them in the Golgi and endoplasmic reticulum, preventing their transport to the cell surface, and allowing infected cells to escape CD8^+^ T-cell recognition [35]. Additionally, E5 limits the MHC I antigen repertoire by affecting the immunoproteasome [36] and suppresses the antiviral type I interferon response by binding to STING and MAVS, thereby restricting their functions [36]. The E6 and E7 oncoproteins alter innate immune responses largely by deregulating interferon functions and interferon-stimulated genes (ISGs) expression. For instance, E7 binds to IRF3 and induces its proteasomal degradation. Furthermore, E7 inhibits the nuclear translocation of p48, a component of the ISGF3 transcription complex, which consists of STAT1, STAT2, and p48, thereby impairing IFNα production [37]. E7 also antagonizes the cGAS-STING pathway, preventing IFNβ expression [38]. On the other hand, E6 interacts with TYK2, preventing the phosphorylation of STAT1 and STAT2, which reduces the expression of ISGs [39]. Both E6 and E7 suppress innate immune activity by downregulating TLR-9 gene expression; specifically, E7 recruits the histone deacetylase HDAC1 and the histone demethylase JARID1B, inducing epigenetic silencing of TLR-9 [40,41].

Immune evasion mechanisms during persistent infection facilitate the establishment of the tumor phenotype. Moreover, in established malignant tumors, the different components of the immune system can further influence tumor progression. Therefore, understanding the mechanisms underlying the maintenance and progression of HPV-positive tumors—particularly those involving interactions with components of the immune system—is essential for the development of therapeutic strategies targeting immune cells and their products.

## 3. Mast Cell Biology and Its Role in HPV-Susceptible Tissues

Mast cells are present in all vascularized tissues in mammals [42]. They have been identified as the initiators of Immunoglobulin E (IgE)/Antigen (Ag)-induced Type I hypersensitivity reactions and, in consequence, they are the target of numerous therapies against allergies. Besides allergic responses, MCs exert protective functions in defense against helminths and bacteria, and participate in wound healing and the inactivation of venoms [42].

MCs originate from precursors that arise from the yolk sac and the aorta-gonad-mesonephros in the initial steps of embryonic development. Later, those progenitors migrate to the fetal liver and, from there, colonize peripheral tissues. In posterior developmental stages, MCs are continuously produced from bone marrow (BM) and migrate as immature precursors to the tissues where they acquire specific characteristics and exert effector functions [43]. In mice, BM-derived MCs and YS-derived MCs coexist in some tissues, suggesting a very long lifetime of specific MC subtypes. A cross-organ analysis of nine prenatal tissues using single cell RNA sequencing (scRNA-seq) revealed that macrophages, mast cells, and NK cells, although present in the first stages of development, acquire immune-effector functions between 10 and 12 weeks post-conception [44]. The transcriptomic signature before that time suggests a role of these cell types in tissue morphogenesis and angiogenesis.

Early studies classified MCs from human and adult mice according to their granular protease content. In mice, two main types of MCs have been identified: 1) the connective tissue mast cells (CTMCs) that express tryptase and chymase; and 2) the mucosal-type mast cells (MMCs) that contain tryptase. In humans, MCs have been classified as tryptase-positive MCs (MCT) and chymase- and tryptase-positive MCs (MCTC) [45]. Histologically, MCT localize in mucosal and epithelial surfaces, nerves, blood vessels, and intestinal mucosa, whereas MCTC are present in subcutaneous tissues, skin, and synovium.

MCs populate the human uterus, but the characteristics of uterine MCs remain insufficiently defined. Early studies reported numerous MCs in human myometrium uniformly distributed throughout the tissue, and fewer in the endometrium [46]. When co-cultured, endometrial cells and MCs interacted by a loop involving the production of the CCL8 chemokine and the activation of the CCR1 receptor [47]. Also, estrogen-mediated activated MCs have been associated with endometriosis [48]. Regarding their physiological functions, uterine MCs respond to estrogens, histamine, and labor-related hormones, such as oxytocin, supporting the idea that they play an important role in human pregnancy, labor, and delivery [49,50].

MCs can also be found in tissues from the oral cavity and oropharynx, structures that are exposed to environmental and internal irritation. Due to these circumstances, these structures are normally affected by reactive, inflammatory, and neoplastic pathologies in which MCs could have an important role. Information about the functional characteristics of MCs in oropharynx tissues is dispersed, although interesting data have been obtained. Tonsillar MCs (ToMCs) express markers such as CD46, CD44, CD54, CD55, CD59, CD43, CD9, and the high affinity IgE receptor (FcεRI), sharing phenotypic traits with lung and uterine MCs rather than skin MCs. Their expression of viral-binding molecules (e.g., CD46, CD54, and CD59) suggests functions in antiviral defense [51]. Regarding the reactive hyperplasic lesions in the oral cavity, MC numbers correlate with inflammation intensity [52]. In oropharyngeal neoplastic processes, MC numbers have been associated with angiogenesis and vascular development [53]; however, their impact on prognosis is contradictory, since elevated numbers of MCs in squamous cell carcinoma have been related to improved survival [54] or increased IL-33 expression and poorer outcomes [55].

As regulators of innate immune protective responses and active participants in inflammation-driven diseases, MCs seem to play multiple roles depending on their origin and the microenvironmental conditions of the tissue where they reside or are recruited. Their location in HPV-susceptible tissues make them an essential cell type to analyze to understand the protective and deleterious inflammatory reactions involved in HPV-induced neoplastic lesions.

## 4. Types of Mast Cell Activation

MCs contain cytoplasmic granules filled with pre-formed inflammatory mediators, such as biogenic amines (histamine, serotonin), proteases (chymase, tryptase), cytokines (tumor necrosis factor alpha, TNF-α), hydrolytic enzymes (β-hexosaminidase, β-galactosidase), and other compounds attached in a heparan sulphate matrix. MCs also possess the enzymatic machinery for the de novo synthesis of bioactive lipids (prostaglandins, leukotrienes, thromboxanes) and contain active signaling pathways that are able to transcribe a plethora of cytokines and growth factors genes, such as interleukin (IL)-2, IL-3, IL-4, IL-10, TNF-α, transforming growth factor (TGF)-β, vascular endothelial growth factor (VEGF), fibroblast growth factor (FGF), nerve growth factor (NGF), and others in response to different stimuli [56,57,58,59,60,61]. Furthermore, the respective proteins are excreted mainly through three distinct mechanisms which involve specific events: (1) anaphylactic degranulation (rapid Ca^2+^-dependent granule fusion); (2) piecemeal degranulation (the progressive release of granule content, maintaining the integrity of the granule membrane); (3) constitutive secretion (protein secretion in vesicles from the Golgi apparatus) [62].

**Figure 1:**
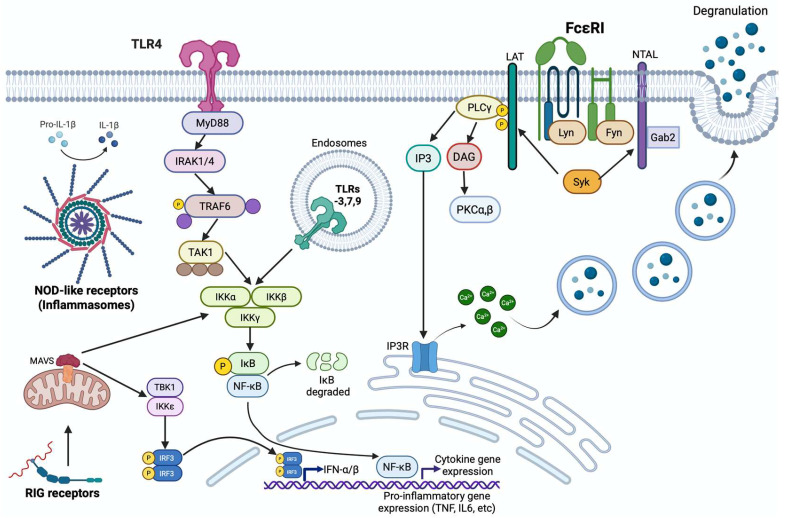
Mast cell activation. MCs can be activated through innate immune receptors like TLRs, NLRs, or RLRs. TLRs and RLRs lead to the activation of NF-κB transcription factor and the synthesis of pro-inflammatory mediators like TNF and IL-6. NLRs lead to the formation of inflammasomes and the production of IL-1β. In addition, MCs can be activated through the high-affinity IgE receptor (FcεRI) which induces the production of the second messengers IP3 and DAG. IP3 binds to its receptors, located in the endoplasmic reticulum, generating the increase of intracellular Ca^2+^ and cell degranulation. See text for more details. Created in BioRender. González Espinosa, C. (2025) https://BioRender.com/67qxovm (accessed on 11 December 2025).

Studies on the physiology of MCs have shown that these cells express a collection of receptors connected to the selective secretion of pro-inflammatory, pro-angiogenic, and regulatory mediators through the mentioned mechanisms. Those classical studies, together with proteomic and transcriptomic analysis of MC populations, have demonstrated that those cells express antibody receptors (to recognize type G or type E immunoglobulins), G protein-coupled receptors (GPCRs), growth factor receptors (i.e., VEGFR1 and 2) [63,64], and diverse pattern-recognition receptors (PRRs), such as Toll-like receptors (TLRs), nucleotide-binding and oligomerization (NOD)-like receptors (NLRs), RIG-I-like receptors (RLRs), C-type lectin receptors (CLRs), the stimulator of interferon genes (STINGs), and others [65,66]. Signaling pathways connecting those molecules with the production of pro-inflammatory mediators, phenotypic adaptations, cytoskeletal changes, and metabolic alterations in MCs constitute a field of intense research due to the relevance of this cell type in protective and deleterious immune reactions. Figure 1 shows the better-studied mechanisms of MC activation.

The best-known activation mechanism of MCs is dependent on the cross-linking of the high affinity immunoglobulin (Ig) E receptor (FcεRI) mediated by IgE/antigen (Ag) complexes. Cross-linked by the recognition of at least two IgE/Ag complexes, the receptor leads to the activation of several intracellular pathways. One intracellular pathway includes mitogen-activated kinases (MAPKs), leading to anaphylactic degranulation, the synthesis of bioactive lipids, the modification of specific mRNAs half-life and the activation of transcription factors for cytokine production, among other important changes in MC epigenetics and metabolism (Figure 1) [67]. The other pathway modulates the activity of the phosphatidylinositol-3-kinase (PI3K) which controls MC survival by the activation of AKT and the production of bioactive lipids and several chemokines, such as CCL2 [68]. In addition, FcεRI controls the activity of the γ isoform of phospholipase C (PLCγ), which produces diacylglycerol (DAG) and phosphatidylinositol 1,4,5 triphosphate (IP3). Those second messengers lead to calcium mobilization outside the endoplasmic reticulum, the opening of calcium membrane channels, and the activation of the classical isoforms of the protein kinase C (PKC). Both events lead to secretory granule membrane fusion with plasma membrane and the release of granules content. The activation of the β isoform of PKC is involved in the formation of a complex formed by the mucosa-associated lymphoid tissue 1 protein (MALT1) and the B-cell lymphoma/leukemia 10 protein (BCL-10). This MALT-1/BCL-10 complex activates the inhibitory-κB kinase (IKK) to allow the translocation to the nucleus of the transcriptional factor NF-κB and the initiation of cytokine gene transcription, which results in cytokine secretion [69].

Besides FcεRI receptors, evidence suggests that the important role that MCs exert in innate immunity is mediated by the activation of distinct Pattern Recognition Receptors (PRRs). As mentioned before, MCs express TLRs, NLRs, and RLRs. So far, all studies conducted on the presence of TLRs in MCs have demonstrated the expression of various TLR family members across different types of MCs [70]. Plasma membrane TLRs typically operate through two distinct mechanisms, both of which rely on TIR domain-containing adaptors that are recruited to TLRs: myeloid differentiation primary response gene 88 (MyD88) and TIR domain-containing adaptor-inducing interferon (IFN)-β [TRIF or Toll-like receptor adaptor molecule 1 (TICAM-1)]. Interestingly, various TLRs can activate the MyD88 signaling pathway, except for TLR3; however, both TLR3 and TLR4 can trigger the TRIF signaling pathway. The signaling cascade that activates nuclear factor kappa B (NF-κB) ultimately results in the expression of various cytokines in the responding cell (Figure 1). On the other hand, the activation of IFN regulatory factors (IRF) 3 or IRF7 leads to the production of type I IFNs. Independent of the adapters utilized, TLR receptor triggering leads to the activation of serine-threonine kinases (IRAKs), ubiquitin ligases (such as TRAF6 and TRAF3), MAPK (such as ERK1/2, JNK and p38), and the inhibitor of NF-κB kinase (IKK), which activates NF-κB [71] (Figure 1).

Another important family of PRRs are the NOD-like receptors. These intracellular molecules perform self-oligomerization after ligand binding to generate complex structures called inflammasomes [72] (Figure 1). Inflammasome aggregates are responsible for the processing of pro-IL-1β and pro-IL-18, together with the proteolysis of gasdermin, which forms pores for cytokine secretion and pyroptosis. Inflammasomes can also promote the nuclear translocation of distinct transcription factors to induce cytokine gene transcription.

To respond to viruses and foreign nucleic acids, MCs are equipped with a battery of sensors that include RIG-like receptors (RLRs) [73]. These molecules are DExD/H box RNA helicases that recognize viral RNA. All RLRs contain a central helicase domain with ATPase activity and an RNA recognition domain in the C-terminal region. Some RLRs, such as RIG-1 and MDA5, possess N-terminal CARDs for signaling. These domains form CARD-CARD complexes after the recognition of modified RNA molecules. These complexes bind to the mitochondrial protein MAVS, leading to the activation of TRAF-2, TRAF-6, TRADD, TRAF-3, and TANK. This finally activates TBK1 and IKK-ε leading to interferon production. Also, pro-inflammatory cytokine synthesis is promoted by the activation of NF-κB (Figure 1).

## 5. Mast Cells in the Tumor Microenvironment (TME)

Nowadays, it is widely accepted that the TME influences different processes such as tumor maintenance and growth, invasion and metastasis, sensitivity to drugs, responsiveness to treatment, and tumor eradication [58]. Regarding immune cells in the TME, although lymphocytes have been traditionally emphasized, increasing evidence highlights the presence of tumor-infiltrating myeloid cells (TIMs) as key regulators of tumor progression. TIMs include plasmacytoid dendritic cells, conventional dendritic cells, monocytes, macrophages, and MCs [74]. Particularly, the presence of MCs in different types of cancer has been demonstrated [74], supporting the belief that these immune cells are important players in the TME with context-dependent, pro- and anti-tumorigenic properties [57].

**Figure 2:**
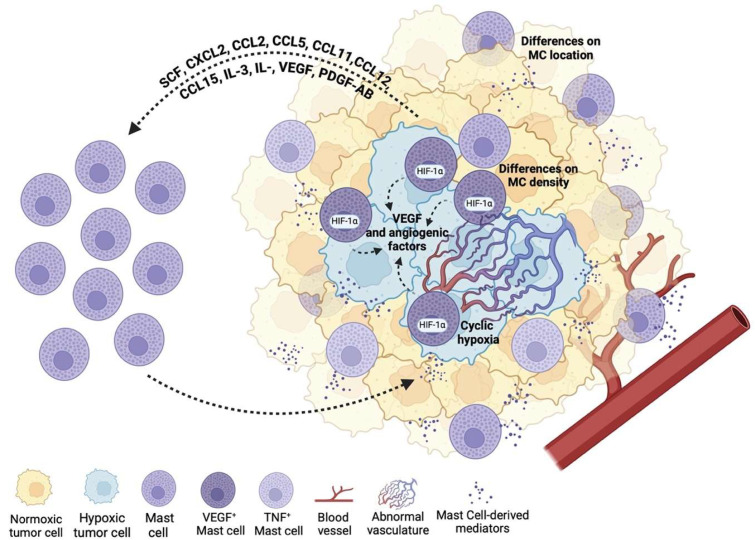
Mast cells influence the tumor microenvironment. Tumor cells secrete different mediators that attract MCs towards the tumor. The number and location of MCs in the tumor may impact their pro- or anti-tumorigenic actions; for example, MCs can be located within hypoxic tumor zones where they release VEGF and other angiogenic factors, promoting aberrant blood vessel formation and cyclic hypoxia which may favor tumor progression. In addition, other MC phenotypes, such as TNF+ MCs, can also be located within tumors, promoting immune responses associated with anti-tumor actions. Created in BioRender. González Espinosa, C. (2025) https://BioRender.com/67qxovm (accessed on 11 December 2025).

The attraction of MCs to a tumor requires the action of different chemokines produced by the tumor cells such as the stem cell factor (SCF), CXCL2, CCL2, CCL5, CCL11, CXCL12, CCL15, IL-3, and IL-33 [58,61]. Moreover, tumor-produced angiogenic factors like the VEGF, platelet-derived growth factor AB (PDGF-AB), and basic fibroblast growth factor (bFGF) also contribute to MC recruitment. In most tumor types, MCs, normally named tumor-associated MCs (TAMCs), can infiltrate solid tumors, or be located in the surrounding tissue, generally around vessels. These differences in MC locations, as well as differences in density, tumor type, and the microenvironment surrounding the cells, impact MCs’ pro- and anti-tumoral properties [75] (Figure 2).

Regarding the impact of MC density on pro- or anti-tumorigenic actions, cancer-type-specific patterns have emerged. It was demonstrated that low MC infiltration in breast cancer is related to increased malignancy and lung metastasis [76]. In contrast, in cervical carcinoma [17], colorectal cancer [77], and esophageal cancers [78], a low MC density within the tumor appears beneficial for patients’ overall survival. Regarding MC localization, in samples of muscle-invasive bladder cancer, high MC infiltration in stromal but not in epithelial tissues correlated with worse clinical outcomes [79]. In addition, in patients with prostate cancer, the enrichment of MCs in benign peri-tumoral regions is associated with an increased recurrence risk and metastasis after prostatectomy [80,81]. Moreover, functional divergence has been demonstrated, where intra-tumoral and peri-tumoral MCs act as negative and positive regulators of tumor growth, respectively [82].

To better understand the duality of MCs’ actions in cancer, an effort to classify these cells in pro- and anti-tumoral cells has been performed according to cytokine production. A recent study demonstrated that the presence of TNF+ or VEGF+ MCs varies across different types of cancer. In general, VEGFA+ MCs are more frequent than TNF+ MCs in most tumors, and when TNF+ MCs predominate, this pattern is associated with a better prognosis. This supports the idea that TNF-containing MCs have anti-tumor properties, while VEGFA+ MCs tend to favor pro-tumoral effects [74]. This functional dichotomy aligns with the known activities of these mediators: where TNF supports cytotoxic immune responses associated with anti-tumor effects, VEGFA is related to angiogenesis.

Specific environmental conditions within the tumor may impact MC VEGF production and other cytokines production. One common condition that modulates MC angiogenic factors production and pro-tumoral effects is hypoxia. Hypoxia commonly arises in solid tumors due to aberrant vasculature and high proliferative demand. In murine models, after MCs infiltrate solid tumors, hypoxic conditions promote an angiogenic switch characterized by the upregulation of pro-angiogenic mediators [83], which appear to be relevant during the early stages of tumor growth [84]. This angiogenic switch in MCs results from hypoxia-driven transcriptional reprogramming mediated by the transcription factor HIF1-α, leading to increased secretion of pro-angiogenic molecules like VEGF-A and CCL-2 [83]. In addition, MCs also produce other pro-angiogenic molecules such as VEGF-B, heparin, histamine, SCF, proteases, and chymases, and pro-lymphangiogenic factors like VEGF-C and -D, which contribute to neovascularization and metastatic dissemination (Figure 2).

The production of pro-angiogenic factors in tumors promotes neovascularization to restore nutrient and oxygen supplies that are indispensable for the growth of malignant tumors; however, tumor-associated angiogenesis often generates aberrant and dysfunctional vasculature that leads to periods of hypoxia/re-oxygenation, also known as cyclic hypoxia (Figure 2). An in vitro study carried out on bone marrow-derived mast cells (BMMCs) showed that cyclic hypoxia changes MCs’ transcriptional profile, demonstrating the ability of these cells to adapt to a changing environment. Interestingly, the authors found that the enrichment of FcεRI produces hyperreactivity to antigen stimulation and an increase in β-hexosaminidase release and *Il-4*, *Il-2*, and *Tnf-α* expression [61], thereby reshaping the TME and potentially enhancing pro-tumoral MC activity.

In general, it is well accepted that MC activation within a tumor leads to extracellular matrix degradation through protease and chymase release, which facilitates invasion and metastases. However, MCs also produce other mediators with additional regulatory roles. A broad repertoire of cytokines, chemokines, lipid mediators, and proteases released by MCs participate in modulating tumor immunity, angiogenesis, and matrix remodeling [85,86,87].

## 6. Mast Cells in HPV-Induced Cancer

### 6.1. Mast Cells in Cervical Cancer

**Figure 3:**
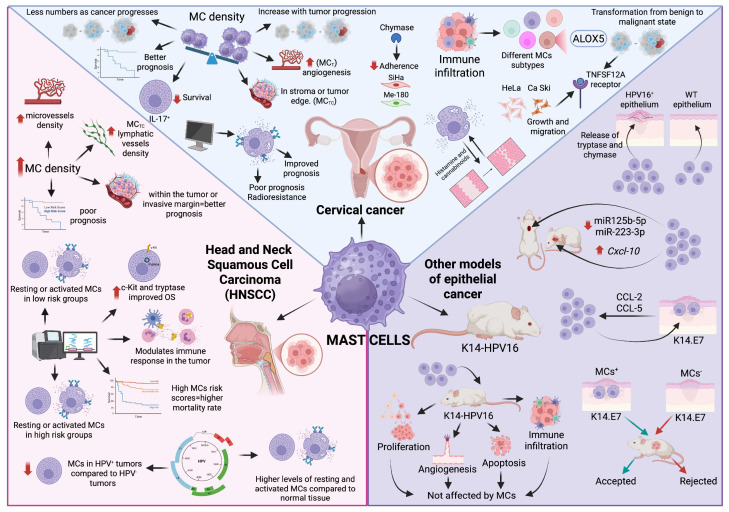
Mast cells in HPV-related cancers. The participation of MCs has been described in HPV-related Cervical Cancer and Head and Neck Squamous Cell Carcinoma. Moreover, murine models of epithelial cancer using K14-HPV16 mice have also shown that MCs play a role in HPV-related cancers. The main findings described in this review are illustrated above. Created in BioRender. González Espinosa, C. (2025) https://BioRender.com/67qxovm (accessed on 11 December 2025).

The study of MCs in CC has been of special interest to distinct authors (Figure 3). In general, most analyses have attempted to associate the number and types of MCs present in the CC tissues with pro- or anti-tumoral effects. To our knowledge, the first study linking MC numbers to CC development dates from the mid-1960s, when Graham and Graham reported that the number of MCs decreases as cancer progresses. Consistently, Kalyani et al. (2016) [88] found a progressive reduction in MC numbers in mild to severe dysplasia, with the lowest numbers in cervical squamous cell carcinoma (CSCC). Conversely, MCs were particularly abundant in biopsies from inflammatory diseases, suggesting that MCs play an active role in inflammatory conditions, such as polypoidal endocervicitis, but not in CC [88]. In line with these observations, a histopathological evaluation of MCs in normal, premalignant, and malignant cervical tissues showed that MC density was highest in an inflamed cervix, decreased in high-grade squamous intraepithelial lesions, and was lowest in invasive carcinoma. MCT predominated across all tissue types, whereas chymase-positive MCs were scarce [89].

Supporting this notion, a recent immunological analysis of IL-17-producing cells in CSCC showed that MCs constitute a substantial subset of IL-17-expressing immune cells in the TME. IL-17 is a pivotal cytokine in cervical carcinogenesis that can modulate inflammation, shape the composition of TME, and influence anti-tumor immunity. Although IL-17 has been associated with both pro- and anti-tumoral effects depending on the producing cell type, its impact is highly context-dependent. In this study, approximately 40% of all MCs produced IL-17, representing about one-quarter of all IL-17-positive cells. Importantly, patients with the lowest MC counts exhibited significantly reduced disease survival, including those with early-stage disease, suggesting that MCs may contribute to a more favorable immune response compared to other IL-17-producing cells [90]. These findings point to a potentially protective or anti-tumoral role of MCs in CC.

Nevertheless, other studies have reported contradictory data. An analysis of normal, dysplastic, and CSCC biopsies showed that MCs increased during tumor progression, correlating with an increase in blood vessels, suggesting that MCs produce angiogenic factors that may contribute to cancer evolution [91]. Concordantly, a positive correlation between the number of MCT cells and blood vessels in cervical intraepithelial neoplasia 2 (CIN2) and carcinoma in situ (CIS) was also demonstrated, suggesting pro-tumoral effects for MCs [92]. Mondal et al. (2014) [93] further demonstrated an increase in MC density from normal cervix to CIS, microinvasive carcinoma, and invasive squamous cell carcinoma. MCs localized mainly at the epithelial/stromal interface and around microvessels. Their findings reinforce the notion that MCs enhance tumor-associated angiogenesis during cervical carcinogenesis [93].

In a study evaluating the involvement of MCs at different locations within normal, low-, and high-grade premalignant lesions and CC samples, MCs were widely distributed in the stroma and near blood vessels in benign lesions and the normal tissues of the uterine cervix [94]. No significant differences were found in the number of MCs at the epithelium/stroma interface in CIN1, CIN2, and CIS when compared with normal tissue. However, a higher proportion of MCs was observed in CIN3 and invasive cancer. Although no changes were found in the number of MCs in the stroma in normal tissue, for CIN1-3 and CIS, the number of these immune cells was higher in invasive cancer compared to normal tissue. However, when comparing stromal MCs with those located at the epithelium/stroma interface, MCs were significantly more abundant in the stroma at all stages except CIS. The authors also identified MCT as the predominant phenotype across cervical tissues, and it was particularly elevated in invasive carcinoma, suggesting that these immune cells may exert a pro-tumoral effect. Additionally, MCTCs seemed to localize at the tumor edge and were not found within the tumor [94]. Concordantly, another immunohistochemical analysis of SCC samples reported that MCTC were located in the peri-tumoral stroma [95].

Furthermore, Guo et al. (2022) [17] studied MC infiltration in CC to determine whether these immune cells may serve as prognostic markers. Using TCGA analyses and tryptase immunostaining of CC tissues, the authors found that low MC infiltration was associated with a better prognosis as well as increased overall survival, which was validated in an independent cohort. The latter was explained by the observation that CC-infiltrating MCs significantly correlated with the presence of CXCL8, IL-1α, IL-1β, CSF2, and CCL20-cytokines and chemokines that may lead to tumor progression [17].

Recent computational and transcriptomic studies consistently highlight the prognostic significance of MC biology in CC, although their proposed roles vary across analytical models. A CIBERSORT-based analysis [96] identified activated MCs as independent predictors of poor overall survival and incorporated them—together with activated memory CD4^+^ T cells and activated NK cells—into a component of a high-risk prognostic model. Similar findings were reported in a radiotherapy-focused immune-risk scoring model [97] where activated MCs were enriched in a high-risk group, suggesting that MCs can be used to indirectly indicate prognosis and radioresistance. Concordantly, a study by Huang et al. (2025) [98] showed that high RFC4 (Replication factor C subunit 4) levels in CC, which are associated with a better prognosis, negatively correlate with activated MC infiltration, reinforcing the idea that activated MCs have pro-tumoral effects. Moreover, Wang et al. (2024) [99] developed an Immune-Related Gene Prognostic index (IRGPI) to stratify CC patients according to survival risk. They found that activated MCs predominated in the high-risk group, whereas resting MCs predominated in the low-risk group.

In contrast, a large immune-deconvolution study [100] reported that activated MCs were enriched in low-risk patients and associated with an improved prognosis, whereas resting MCs were enriched in high-risk patients (worst prognosis), suggesting that MC activation may support anti-tumor immunity rather than tumor progression. Collectively, these bioinformatic findings demonstrate that MCs are important immunologic determinants of prognosis in CC, yet their functional impact appears context-dependent. While several models link activated MCs to high-risk disease and immunosuppression, others associate them with improved survival, suggesting potential anti-tumor roles. This variability underscores that MC function likely depends on their activation state and the surrounding immune microenvironment, warranting further mechanistic investigation.

Recently, using scRNA-seq technology, it was confirmed that MCs infiltrate CC and that seven different MC subpopulations exist within cervical tumors, varying according to lesion type. One MC subpopulation, termed ALOX5+ (due to its high arachidonate 5-lipoxygenase expression), was particularly abundant in lesions associated with progression to CC, suggesting that these MCs may play an important role in the transition from benign to malignant states through the regulation of immunity and inflammatory responses. The transformative role of ALOX5+ MCs relies on activation of the TWEAK signaling pathway through the TNFSF12A receptor, which is associated with cell migration and invasion. The latter was supported by in vitro experiments using HeLa and Ca Ski cells, where the downregulation of TNFSF12A suppressed CC growth and migration. These finding indicate that ALOX5+ MCs may serve as a potential therapeutic target in CC patients [101]. It is also worth noting that the identification of ALOX5 as a potential marker for pro-tumoral MCs suggests that, in addition to cytokines and growth factors, leukotrienes and arachidonic acid-derived mediators are key regulators of a tumor-favorable TME. However, despite providing novel insights, the work remains mostly exploratory, with functional validation limited to in vitro assays, without a direct assessment of MC-specific mechanisms or in vivo confirmation of the TWEAK-TNFSF12A axis.

A single cell RNA-sequencing analysis of CC tissues also revealed changes in MCs and other immune populations in the TME after radiotherapy [102]. The results revealed that after radiotherapy MCs emerge as one of the most enriched cell populations. Radiotherapy also activated specific MC subclusters characterized by the upregulation of classical activation and immunosuppressive genes. In addition, the authors found that MCs engaged in pathways related to lipid mediators’ synthesis, angiogenesis, cytokine release, and extracellular matrix remodeling, which contributes to an immunosuppressive and pro-tumorigenic microenvironment. A cell–cell communication analysis indicated that MCs strengthen interactions with macrophages and other myeloid cell populations, promoting VEGF- and TGF-β-mediated angiogenesis, tissue repair, and immune evasion. Collectively, these findings showed MCs as dynamic modulators of the TME after radiotherapy which can be related to poor prognosis and serve as potential targets for improving treatment responses in CC [102]. Although the results are interesting, the study presented some limitations as the authors only analyzed a small sample size and it was based only on transcriptomic data, without functional in vitro or in vivo validation. In addition, further mechanistic studies are required to confirm MCs’ contribution and therapeutic relevance.

In addition to MC density and subtypes, the pro-and anti-tumoral effects of MC-derived mediators in CC have also been explored. For example, an in vitro study using SW756 CC cells (HPV18 positive) demonstrated that LAD2 human MCs promote CC cell migration through the release of histamine and cannabinoids. In addition, the authors showed that communication between MCs and CC cells was bidirectional, as SW756 cells induced MC degranulation, an effect that was inhibited by cannabinoids acting through CB2 receptors [103]. Moreover, Diaconu et al. (2011) [95] found that recombinant chymase reduced the adhesion of SiHa (HPV16 positive) and ME-180 (HPV68 positive) cells, while normal keratinocytes underwent apoptotic cell death. This suggests that MC-derived chymase may contribute to pro-tumoral effects by promoting cancer cell detachment and spreading. However, although recombinant chymase reduced adhesion in these cell lines and induced apoptosis in keratinocytes, these findings are derived from a single in vitro study and may not accurately reflect in vivo conditions. The concentrations of recombinant chymase used experimentally (5 μg/mL) are not known to occur physiologically, and there is no current evidence that endogenous MC-derived chymase reaches comparable levels in the TME. Therefore, in vivo confirmation and mechanistic studies are necessary to determine whether MC-derived chymase contributes to CC progression under physiological conditions.

### 6.2. Mast Cells in Head and Neck Squamous Cell Carcinoma

Head and Neck Squamous Cell Carcinoma (HNSCC) is the seventh most prevalent cancer in humans [104]. This type of cancer comprises a group of malignancies that affect mucosal linings from the nasopharynx, oral cavity, salivary glands, oropharynx, hypopharynx, and larynx [105]. Nowadays, HPV infection represents a major risk factor for this cancer, accounting for approximately 25% of all cases [4,18].

Several studies have described the participation of MCs in HNSCC (Figure 3), although most do not differentiate between the HPV-positive and HPV-negative tumors. Nonetheless, we cannot exclude the findings of these studies, since a considerable proportion of head and neck cancers are HPV-driven. Laishram et al. (2017) [106], reported a positive correlation between MC density (MCD) and microvessel density (MVD) in normal oral tissue, premalignant lesions, and oral squamous cell carcinoma (OSCC), with both MCD and MVD significantly higher in OSCC. In another study of OSCC samples, MCTC density was higher in patients with nodal metastasis when compared with those without nodal metastasis. Moreover, MCTC density was significantly associated with histological grade, local progression, clinical stage, MVD, and lymphatic vessels density (LVD). Although attempts to correlate MCTC density with prognosis did not reach statistical significance, the cases with higher MCTC densities tended to show poorer outcomes. The authors suggested that OSCC progression may depend on chymase-mediated activation of MIA (melanoma inhibitory activity) and MIA2, promoting angiogenesis and lymphangiogenesis [107]. Tzorakoleftheraki and Koletsa (2024) [108] performed a systematic review showing that MCs in either active or resting states, linked to IgE receptor activation [109], were associated with a worse prognosis in HNSCC.

In 2022, Cai et al. [110], using scRNA-seq, developed a gene signature for MCs in HNSCC composed of nine genes (KIT, RAB32, CATSPER1, SMYD3, LINC00996, SOCS1, AP2M1, LAT, and HSP90B1) that accurately stratified patient survival outcomes and functioned as an independent prognostic factor. Higher MC risk scores correlated with a higher mortality rate. Differential gene expression analysis revealed that the high-risk group exhibited deregulation of the genes involved in immune responses and cell-mediated immunity and in the low-risk MC score groups, the authors found that the former contained deregulated genes associated with immune response and cell-mediated immunity. This indicated that MCs modulate inflammatory responses within the TME. Consistent with this finding, another bioinformatic study identified a nine-gene signature related to immune infiltration (MORF4L2, CTSL1, TBC1D2, C5orf15, LIPA, WIPF1, CXCL13, TMEM173, and ISG20) with prognostic value in patients with HNSCC. Patients in the groups with high scores were associated with poor clinical outcomes. Interestingly, when evaluating cell subpopulations in HNSCC, an increase in activated MCs was found in the high-risk group [111]. A more recent analysis integrating scRNA-seq and bulk RNA-seq identified a 14-gene MC-related prognostic signature, in which low-risk patients, characterized by higher resting MC signatures, exhibited better survival and immune infiltration. In contrast, activated MC-related signatures were predominantly enriched in the high-risk group, indicating their association with poorer clinical outcomes [112].

Additionally, a four-gene aging-related signature (BAK1, DKK1, CDKN2A, and MIF) in tumor tissues was shown to predict poor outcomes in HNSCC. Patients with a high-risk score, and patients in the high-risk group were found to have higher resting MC infiltration and lower activated MC infiltration than patients in the low-risk group [113]. These findings support the concept that an aging microenvironment has a major impact on tumor progression.

In contrast with the predominantly unfavorable associations described above, some studies suggest beneficial roles for MCs in HNSCC. In OSCC, a high density of MCs located within the tumor or at the invasive front was associated with a better prognosis, a finding supported by evidence showing that patients with elevated MC infiltration, mainly in the invasive margin, exhibit significantly lower recurrence rates. Consistent with this finding, an analysis of an independent TCGA HNSCC cohort revealed that a higher expression of MC-related markers, including c-Kit and tryptases, correlated with improved OS [114].

In addition to these histological and clinical observations, transcriptomic analyses further support the context-dependent role of MCs in HNSCC [115]. A recent study using CIBERSORTx to quantify tumor-infiltrating immune cells across multiple HNSCC subsites showed that resting MCs were associated with improved survival, particularly showing better DFS in hypopharyngeal tumors and improved DFS and OS in oral cavity cancers. In contrast, activated MCs were associated with a worse prognosis (reduced DFS and OS) in the overall cohort analysis [115]. However, the study presents several limitations that should be considered when interpreting these results. For example, the dataset does not stratify patients by HPV status, limiting the ability to distinguish MC-specific effects in HPV-positive or HPV-negative HNSCC. Additionally, validating these computational findings with experimental methods like IHC-based MC quantification or the exploration of mechanistic pathways linking MCs to prognosis could help strengthen the results or explain why resting and activated MCs have opposite prognostic effects, respectively.

Overall, current evidence demonstrates that MCs exert contrasting and context-dependent effects in HNSCC. Therefore, the prognostic significance of MCs in HNSCC is not uniform but instead depends on different characteristics such as activation state, special distribution, functional phenotype, and the biological context of each tumor. These observations demonstrate the need for more studies to determine the precise role of MCs in this type of cancer.

Despite the mentioned studies, little information exists regarding the role of these cells when the origin of this cancer is related to HPV (Figure 3). Zhou et al. (2021) [116] reported that, compared to normal tissue, HPV-positive HNSCC exhibited higher levels of resting and activated MCs, whereas HPV-negative tumors showed higher levels of resting MCs but lower levels of activated MCs. Recently, Tosi et al., (2022) [117] analyzed the tumor immune microenvironment (TIME) in oropharyngeal squamous cell carcinoma and found that HPV-positive tumors were enriched in tumor infiltrating lymphocytes (TILs) and check point molecules such as PD-L1, compared to HPV-negative tumors. Interestingly, patients whose HPV-positive tumors had high infiltration of TILs exhibited a longer period of disease-free survival (DFS). In particular, when evaluating MCs/TILs ratios in HPV-positive and -negative tumors, the proportion of MCs was lower in the HPV-positive tumors. Moreover, in the HPV-negative tumors, the higher MCs/TILs ratio was associated with a worse DFS. Concordantly, when evaluating the immune context in HPV-positive and -negative HNSCC tumors, a decrease in MCs was found in the HPV-positive tumors [118]. Overall, current evidence indicates that, in HNSCC tumors, MCs are generally associated with worse clinical outcomes; however, their specific role in HPV-positive HNSCC remains controversial and needs further analysis.

### 6.3. Mast Cells in Other Models of HPV-Induced Epithelial Cancer

In addition to cervical and HNSCC, HPV is also responsible for malignancies in several anatomical regions such as the vulva, vagina, anus, and penis. However, information regarding the presence and role of MCs in anatomical sites other than the cervix and head and neck is scarce, and most available studies focus on skin neoplasia in murine models of HPV-induced cancer. The most widely used model is the transgenic mouse carrying the early region genes of HPV16 (E6 and E7 oncogenes) expressed under the control of a keratin 14 (K14) promoter/enhancer, commonly referred to as K14-HPV16 mice (Figure 3). In this skin cancer model, the mice develop epidermal hyperplasia at one month of age which progress to angiogenic dysplasia between three and six months, and to squamous cell carcinoma by one year [119].

In this murine model, HPV16-harboring epithelia recruit higher numbers of MCs compared to wild type (WT) animals [120,121,122,123]. Notably, MCs infiltrate hyperplastic and dysplastic areas and accumulate at the invasive front of skin carcinomas but remain largely absent from the tumor core. At these peripheral sites, MCs release tryptase and chymase near capillaries and epithelial basement membranes [120], promoting fibroblast proliferation within the stroma and driving hyperplasic skin angiogenesis. Conversely, premalignant angiogenesis is reduced in MC-deficient HPV16 transgenic mice, suggesting that MCs in the premalignant lesions, and in the front of invading cancer, are required for reorganizing stromal architecture and hyperactivate angiogenesis.

Another study in K14-HPV16 mice reported increased MC infiltration in the ear and chest tissues of HPV transgenic mice, when compared to WT controls [123]. Moreover, MC infiltration increased progressively from normal epithelium to hyperplastic/dysplastic epithelium, suggesting that HPV-positive cells may produce mediators that attract MCs and potentially contribute to cancer progression. Reduced expression of miR-125b-5p and miR-223-3p, along with increased *Cxcl10* transcript levels, correlated with MC infiltration in this model [123]. Additionally, in a transgenic model of mice expressing HPV16 E7 (K14-E7 mice), this oncoprotein promoted the epidermal production of CCL2 and CCL5, further promoting MC recruitment to the skin [121,123]. These MCs showed evidence of degranulation, unlike those in non-transgenic skin. Furthermore, using MC-positive and MC-deficient skin grafts expressing E7 in syngeneic immunocompetent mice, the authors showed that the MC-positive E7 grafts were not rejected, unlike the E7 grafts that lacked MCs. These results indicate that MCs exert a local immunosuppressive effect—likely through regulation of CD8^+^ T cell activity—which may favor the persistence of E7-driven premalignant lesions.

Despite evidence suggesting that MCs promote stromal remodeling, immunosuppression, and angiogenesis in HPV-induced skin carcinogenesis, the findings of Ghouse et al. (2018) [122] challenge this interpretation. In a model of K14-HPV16 transgenic mice with transformed skin or inoculated syngeneic tumors (B16, LCC, and B49 cells), the authors found that MCs massively accumulate in HPV-transformed skin and in inoculated tumors. Interestingly, HPV-induced tumor development was not affected in the skin of MC-deficient mice, as cell proliferation, angiogenesis, or apoptosis did not change when compared to the control animals [122]. Also, MC deficiency did not affect the composition of inflammatory infiltrates, or the progression of HPV-transformed skin towards squamous cancer. With this evidence, the authors concluded that skin resident MCs are not required for the development or progression of HPV-induced epidermal neoplasia.

This discrepancy underlines important context-dependent differences in how MCs influence HPV-associated tumor development, which may depend on factors such as tumor stage, tissue microenvironment, or the experimental system employed. Together, these contrasting results demonstrate that additional studies are needed to determine the specific circumstances under which MCs either promote or inhibit the progression of HPV-induced cancer.

## 7. Mast Cells as Potential Therapeutic Targets for HPV-Related Cancers

To the best of our knowledge, no studies have directly explored MCs as therapeutic targets for HPV-related cancer treatments. Therefore, the considerations discussed below remain speculative and are extrapolated from evidence generated in other cancer types, from which we infer how MCs could potentially be explored as therapeutic targets in this context.

Given the involvement of MCs in HPV-induced cancer, these cells may represent potential therapeutic targets. As noted earlier, MC abundance in HPV-positive tumors may contribute to cancer progression; thus, strategies aimed at inhibiting or promoting MC recruitment could potentially improve treatment responses, depending on their functional role in each tumor. For example, several inhibitors of c-kit (the SCF receptor)—including imatinib, mesylate, masitinib, nilotinib, and dasatinib—have shown beneficial effects in other cancers [124,125] by blocking SCF, a key chemoattractant and survival factor for MCs. Conversely, the adoptive transfer of MCs has demonstrated anti-tumor effects in animal models of various cancers [126,127]. These findings indicate that either inhibiting or enhancing MC activity could be therapeutically beneficial, depending on the specific effect of MCs in HPV-related tumors.

The inhibition of MC recruitment may also improve the effectiveness of immune checkpoint blockade (ICB) in HPV-related cancers. ICB therapies target molecules such as the cytotoxic T lymphocyte antigen-4 (CTLA-4), programmed cell death-1 (PD-1), and programmed cell death ligand-1 (PD-L1). Despite their success in several malignancies, PD-1/PDL-1 inhibitors have shown low response rates in clinical trials for HPV-related cancers [128,129]. This limited efficacy may be partly driven by resistance mechanisms involving immune cells, including MCs. Evidence shows that MC infiltration correlates with poor responses to anti-PD-1 therapy in several cancers [130,131,132] and that reducing or stabilizing MC numbers can improve responses to PD-1/PD-L1 inhibition [131,132,133]. Another therapeutic approach could involve blocking PDL-1 expression on MCs or inhibiting the molecules that induce it. For instance, the inhibition of TNF-α, which upregulates PD-L1 expression on MCs contributing to T cell inhibition [134], may be another potentially useful strategy; however, TNF-α blockade may have relevant effects on other immune responses. Overall, these observations support the concept of MC-targeted interventions as potential strategies for HPV-related cancers.

Targeting MC degranulation of MC-derive mediators may also provide therapeutic benefits. MC-stabilizing agents, such as sodium cromoglycate, have shown anti-tumor effects in various models [85,135,136,137,138]. Similarly, inhibitors of MC-derived histamine may also be useful for cancer treatment. In fact, the use of antihistamines, specifically H1-antihistamines, have been associated with reduced MC infiltration and VEGF levels in a murine model of melanoma [139], and with better treatment response in some other cancers [140]. Compounds with mixed activity, such as ketotifen (a membrane stabilizer that blocks calcium channels and acts as an H1 receptor antagonist) have also demonstrated anti-tumor effects [133]. The inhibition of tryptase using agents such as tranilast, nafamostat mesylate, and gabexate mesylate has been shown to reduce angiogenesis and metalloproteinase activity, contributing to anti-tumor effects in several cancers [141]. Although these strategies have not been evaluated in HPV-related tumors, they hold promising potential given the involvement of MCs in these malignancies.

Another potential strategy for HPV-related cancer is to modulate MC secretory profiles to promote anti-tumor immunity through the activation of toll-like receptors (TLRs). For example, the activation of MCs TLR2 with Pam3CSK4 triggers CCL3 and IL-6 secretion and recruits cytotoxic T cells, inhibiting melanoma growth in mice [142]. Similarly, the activation of the TLR7/CCL2 axis in MCs recruits tumor-killing plasmacytoid dendritic cells [143]. Shifting MCs toward a TNF+ rather than VEGF+ phenotype may also be beneficial in HPV-positive cancer treatment; however, this therapeutic strategy needs further investigation.

## 8. Perspectives and Future Directions

Understanding the molecular mechanisms governing the interaction between the TME and tumor cells will provide evidence for understanding the pathogenesis of cancer, as well as providing tools for the development of therapeutic strategies and the identification of molecules with prognostic and predictive value. The TME plays a central role in the progression of HPV-positive tumors, and recent studies have increasingly examined the contribution of MCs to the establishment and maintenance of tumor phenotype. Notably, the close interplay between MCs and other immune, stromal, and cancer cells may influence the immunologically “hot” phenotype characteristic of HPV-positive tumors, and elucidating this relationship could improve therapeutic efficacy in HPV-related cancers.

In tumors with high MC infiltration, therapeutic strategies targeting MC recruitment, degranulation, or MC-derived inflammatory mediators have shown robust efficacy in preclinical models. Therefore, clarifying the specific involvement of MCs in HPV-related tumors could allow the application of these strategies for clinical use. Moreover, intra-tumoral hypoxia is a key event in TME remodeling, where the presence of MCs has been associated with hypoxic areas, favoring the tumor phenotype. Remarkably, CC and HNSCC are characterized by a hypoxic environment that has been associated with low treatment responses and poor outcomes [144,145]. Evaluating the localization of MCs relative to hypoxic regions and blood vessels within the tumor may therefore clarify the participation of these cells in HPV-related tumors and reveal potential therapeutic targets. Additionally, MCs may serve as biomarkers for predicting the clinical outcomes of patients with HPV-positive tumors. In this regard, the analysis and association of MCs’ density, location, or granules content with tumor progression may have clinical relevance.

In CC, high expression of HPV oncogenes is associated with a worse prognosis [146]. This suggests that these oncogenes regulate molecular processes that promote tumor aggressiveness, potentially through TME remodeling. To date, few studies have specifically examined how the HPV oncoproteins E6 and E7 influence MC recruitment in tumors. Uncovering these mechanisms will be essential for understanding the biology of HPV-related cancers, deciphering how MC-derived mediators modulate cancer cell behavior, and defining the impact of MC-tumor interactions on proliferation, migration, invasion, and angiogenesis.

## Data Availability

No new data were created or analyzed in this study.

## Abbreviations

The following abbreviations are used in this manuscript:

TME	Tumor microenvironment
MCs	Mast cells
HPV	Human papilloma virus
LR-HPV	Low-risk Human papilloma virus
HR-HPV	High-risk Human papilloma virus
HNCs	Head and neck cancers
IAPs	Inhibitory of apoptosis proteins
pRb	Retinoblastoma tumor suppressor protein
EGFR	Epithelial growth factor receptor
EGF	Epithelial growth factor
CDK	Cyclin-dependent kinase
ISGs	Interferon stimulated genes
scRNA-seq	Single cell RNA sequencing
CTMCs	Connective tissue mast cells
MMCs	Mucosal mast cells
MC_T_	Tryptase positive mast cells
MC_TC_	Tryptase and chymase mast cells
ToMCs	Human tonsillar mast cells
TNF-α	Tumor necrosis factor alpha
VEGF	Vascular endothelial growth factor
FGF	Fibroblast growth factor
NGF	Nerve growth factor
GPCRSs	G protein-coupled receptors
PRRs	Pattern-recognition receptors
TLRs	Toll-like receptors
NLRs	Nucleotide-binding and oligomerization-like receptors
RLRs	RIG-I-like receptors
CLRs	C-type lectin receptors
STINGs	Stimulated interferon genes
TIMs	Tumor-infiltrating myeloid cells
SCF	Stem cell factor
TAMCs	Tumor-associated mast cells
BMMCs	Bone marrow derived mast cells
CIN1, 2, 3	Cervical intraepithelial neoplasia 1, 2, or 3
CIS	Carcinoma in situ
HNSCC	Head and neck squamous cell carcinoma
MCD	Mast cell density
MVD	Microvessels density
OSCC	Oral squamous cell carcinoma
TIME	Tumor immune microenvironment
TILs	Tumor infiltrating lymphocytes
DFS	Disease-free survival
ICB	Immune checkpoint blockade
PD-1	Programmed cell death 1
PD-L1	Programmed cell death ligand 1

## References

[1] Serrano B., Brotons M., Bosch F.X., Bruni L. (2018). Epidemiology and Burden of HPV-Related Disease. Best Pract. Res. Clin. Obstet. Gynaecol..

[2] Bzhalava D., Guan P., Franceschi S., Dillner J., Clifford G. (2013). A Systematic Review of the Prevalence of Mucosal and Cutaneous Human Papillomavirus Types. Virology.

[3] de Martel C., Plummer M., Vignat J., Franceschi S. (2017). Worldwide Burden of Cancer Attributable to HPV by Site, Country and HPV Type. Int. J. Cancer.

[4] Kreimer A.R., Clifford G.M., Boyle P., Franceschi S. (2005). Human Papillomavirus Types in Head and Neck Squamous Cell Carcinomas Worldwide: A Systematic Review. Cancer Epidemiol. Biomark. Prev..

[5] Carlander A.F., Jakobsen K.K., Bendtsen S.K., Garset-Zamani M., Lynggaard C.D., Jensen J.S., Grønhøj C., Buchwald C.V. (2021). A Contemporary Systematic Review on Repartition of HPV-Positivity in Oropharyngeal Cancer Worldwide. Viruses.

[6] Liao C.-I., Francoeur A.A., Kapp D.S., Caesar M.A.P., Huh W.K., Chan J.K. (2022). Trends in Human Papillomavirus-Associated Cancers, Demographic Characteristics, and Vaccinations in the US, 2001–2017. JAMA Netw. Open.

[7] Li H.-X., Wang S.-Q., Lian Z.-X., Deng S.-L., Yu K. (2023). Relationship between Tumor Infiltrating Immune Cells and Tumor Metastasis and Its Prognostic Value in Cancer. Cells.

[8] Vahidian F., Duijf P.H.G., Safarzadeh E., Derakhshani A., Baghbanzadeh A., Baradaran B. (2019). Interactions between Cancer Stem Cells, Immune System and Some Environmental Components: Friends or Foes?. Immunol. Lett..

[9] Partlová S., Bouček J., Kloudová K., Lukešová E., Zábrodský M., Grega M., Fučíková J., Truxová I., Tachezy R., Špíšek R. (2015). Distinct Patterns of Intratumoral Immune Cell Infiltrates in Patients with HPV-Associated Compared to Non-Virally Induced Head and Neck Squamous Cell Carcinoma. Oncoimmunology.

[10] Gameiro S.F., Evans A.M., Mymryk J.S. (2022). The Tumor Immune Microenvironments of HPV+ and HPV− Head and Neck Cancers. WIREs Mech. Dis..

[11] Zeng P.Y.F., Cecchini M.J., Barrett J.W., Shammas-Toma M., De Cecco L., Serafini M.S., Cavalieri S., Licitra L., Hoebers F., Brakenhoff R.H. (2022). Immune-Based Classification of HPV-Associated Oropharyngeal Cancer with Implications for Biomarker-Driven Treatment de-Intensification. eBioMedicine.

[12] Khazaie K., Blatner N.R., Khan M.W., Gounari F., Gounaris E., Dennis K., Bonertz A., Tsai F.-N., Strouch M.J., Cheon E. (2011). The Significant Role of Mast Cells in Cancer. Cancer Metastasis Rev..

[13] Maciel T.T., Moura I.C., Hermine O. (2015). The Role of Mast Cells in Cancers. F1000Prime Rep..

[14] Dabiri S., Huntsman D., Makretsov N., Cheang M., Gilks B., Bajdik C., Gelmon K., Chia S., Hayes M. (2004). The Presence of Stromal Mast Cells Identifies a Subset of Invasive Breast Cancers with a Favorable Prognosis. Mod. Pathol..

[15] Rajput A.B., Turbin D.A., Cheang M.C.U., Voduc D.K., Leung S., Gelmon K.A., Gilks C.B., Huntsman D.G. (2008). Stromal Mast Cells in Invasive Breast Cancer Are a Marker of Favourable Prognosis: A Study of 4,444 Cases. Breast Cancer Res. Treat..

[16] Malfettone A., Silvestris N., Saponaro C., Ranieri G., Russo A., Caruso S., Popescu O., Simone G., Paradiso A., Mangia A. (2013). High Density of Tryptase-Positive Mast Cells in Human Colorectal Cancer: A Poor Prognostic Factor Related to Protease-Activated Receptor 2 Expression. J. Cell. Mol. Med..

[17] Guo F., Kong W.-N., Li D.-W., Zhao G., Wu H.-L., Anwar M., Shang X.-Q., Sun Q.-N., Ma C.-L., Ma X.-M. (2022). Low Tumor Infiltrating Mast Cell Density Reveals Prognostic Benefit in Cervical Carcinoma. Technol. Cancer Res. Treat..

[18] Sabatini M.E., Chiocca S. (2020). Human Papillomavirus as a Driver of Head and Neck Cancers. Br. J. Cancer.

[19] White E.A. (2019). Manipulation of Epithelial Differentiation by HPV Oncoproteins. Viruses.

[20] Lo Cigno I., Calati F., Girone C., Catozzo M., Gariglio M. (2024). High-Risk HPV Oncoproteins E6 and E7 and Their Interplay with the Innate Immune Response: Uncovering Mechanisms of Immune Evasion and Therapeutic Prospects. J. Med. Virol..

[21] Moody C.A. (2022). Regulation of the Innate Immune Response during the Human Papillomavirus Life Cycle. Viruses.

[22] Vink M.A., Bogaards J.A., van Kemenade F.J., de Melker H.E., Meijer C.J.L.M., Berkhof J. (2013). Clinical Progression of High-Grade Cervical Intraepithelial Neoplasia: Estimating the Time to Preclinical Cervical Cancer From Doubly Censored National Registry Data. Am. J. Epidemiol..

[23] Morgan I.M. (2025). The Functions of Papillomavirus E2 Proteins. Virology.

[24] Porter V.L., Marra M.A. (2022). The Drivers, Mechanisms, and Consequences of Genome Instability in HPV-Driven Cancers. Cancers.

[25] Murakami I., Egawa N., Griffin H., Yin W., Kranjec C., Nakahara T., Kiyono T., Doorbar J. (2019). Roles for E1-Independent Replication and E6-Mediated P53 Degradation during Low-Risk and High-Risk Human Papillomavirus Genome Maintenance. PLoS Pathog..

[26] Zhang Y., Dakic A., Chen R., Dai Y., Schlegel R., Liu X. (2017). Direct HPV E6/Myc Interactions Induce Histone Modifications, Pol II Phosphorylation, and HTERT Promoter Activation. Oncotarget.

[27] Moody C.A., Laimins L.A. (2010). Human Papillomavirus Oncoproteins: Pathways to Transformation. Nat. Rev. Cancer.

[28] Barrow-Laing L., Chen W., Roman A. (2010). Low- and High-Risk Human Papillomavirus E7 Proteins Regulate P130 Differently. Virology.

[29] James C.D., Saini S., Sesay F., Ko K., Felthousen-Rusbasan J., Iness A.N., Nulton T., Windle B., Dozmorov M.G., Morgan I.M. (2021). Restoring the DREAM Complex Inhibits the Proliferation of High-Risk HPV Positive Human Cells. Cancers.

[30] Shin M.-K., Balsitis S., Brake T., Lambert P.F. (2009). Human Papillomavirus E7 Oncoprotein Overrides the Tumor Suppressor Activity of P21Cip1 in Cervical Carcinogenesis. Cancer Res..

[31] Yan X., Shah W., Jing L., Chen H., Wang Y. (2010). High-Risk Human Papillomavirus Type 18 E7 Caused P27 Elevation and Cytoplasmic Localization. Cancer Biol. Ther..

[32] Venuti A., Paolini F., Nasir L., Corteggio A., Roperto S., Campo M.S., Borzacchiello G. (2011). Papillomavirus E5: The Smallest Oncoprotein with Many Functions. Mol. Cancer.

[33] Ilahi N.E., Bhatti A. (2020). Impact of HPV E5 on Viral Life Cycle via EGFR Signaling. Microb. Pathog..

[34] Steinbach A., Riemer A.B. (2018). Immune Evasion Mechanisms of Human Papillomavirus: An Update. Int. J. Cancer.

[35] Kawase K., Taguchi A., Ishizaka A., Lin J., Ueno T., Yoshimoto D., Eguchi S., Mori S., Sone K., Mori M. (2024). Allelic Loss of HLA Class I Facilitates Evasion from Immune Surveillance in Cervical Intraepithelial Neoplasia. HLA.

[36] Miyauchi S., Kim S.S., Jones R.N., Zhang L., Guram K., Sharma S., Schoenberger S.P., Cohen E.E.W., Califano J.A., Sharabi A.B. (2023). Human Papillomavirus E5 Suppresses Immunity via Inhibition of the Immunoproteasome and STING Pathway. Cell Rep..

[37] Barnard P., McMillan N.A. (1999). The Human Papillomavirus E7 Oncoprotein Abrogates Signaling Mediated by Interferon-Alpha. Virology.

[38] Lau L., Gray E.E., Brunette R.L., Stetson D.B. (2015). DNA Tumor Virus Oncogenes Antagonize the CGAS-STING DNA-Sensing Pathway. Science.

[39] Li S., Labrecque S., Gauzzi M.C., Cuddihy A.R., Wong A.H.T., Pellegrini S., Matlashewski G.J., Koromilas A.E. (1999). The Human Papilloma Virus (HPV)-18 E6 Oncoprotein Physically Associates with Tyk2 and Impairs Jak-STAT Activation by Interferon-α. Oncogene.

[40] Hasan U.A., Zannetti C., Parroche P., Goutagny N., Malfroy M., Roblot G., Carreira C., Hussain I., Müller M., Taylor-Papadimitriou J. (2013). The Human Papillomavirus Type 16 E7 Oncoprotein Induces a Transcriptional Repressor Complex on the Toll-like Receptor 9 Promoter. J. Exp. Med..

[41] Pacini L., Savini C., Ghittoni R., Saidj D., Lamartine J., Hasan U.A., Accardi R., Tommasino M. (2015). Downregulation of Toll-Like Receptor 9 Expression by Beta Human Papillomavirus 38 and Implications for Cell Cycle Control. J. Virol..

[42] Valent P., Akin C., Hartmann K., Nilsson G., Reiter A., Hermine O., Sotlar K., Sperr W.R., Escribano L., George T.I. (2020). Mast Cells as a Unique Hematopoietic Lineage and Cell System: From Paul Ehrlich’s Visions to Precision Medicine Concepts. Theranostics.

[43] Gentek R., Ghigo C., Hoeffel G., Bulle M.J., Msallam R., Gautier G., Launay P., Chen J., Ginhoux F., Bajénoff M. (2018). Hemogenic Endothelial Fate Mapping Reveals Dual Developmental Origin of Mast Cells. Immunity.

[44] Suo C., Dann E., Goh I., Jardine L., Kleshchevnikov V., Park J.-E., Botting R.A., Stephenson E., Engelbert J., Tuong Z.K. (2022). Mapping the Developing Human Immune System across Organs. bioRxiv.

[45] Irani A.A., Schechter N.M., Craig S.S., DeBlois G., Schwartz L.B. (1986). Two Types of Human Mast Cells That Have Distinct Neutral Protease Compositions. Proc. Natl. Acad. Sci. USA.

[46] Crow J., More L., Howe S. (1988). The Mast Cells of the Human Uterus. APMIS.

[47] Li T., Wang J., Guo X., Yu Q., Ding S., Xu X., Peng Y., Zhu L., Zou G., Zhang X. (2020). Possible Involvement of Crosstalk between Endometrial Cells and Mast Cells in the Development of Endometriosis via CCL8/CCR1. Biomed. Pharmacother..

[48] McCallion A., Nasirzadeh Y., Lingegowda H., Miller J.E., Khalaj K., Ahn S., Monsanto S.P., Bidarimath M., Sisnett D.J., Craig A.W. (2022). Estrogen Mediates Inflammatory Role of Mast Cells in Endometriosis Pathophysiology. Front. Immunol..

[49] Menzies F.M., Shepherd M.C., Nibbs R.J., Nelson S.M. (2011). The Role of Mast Cells and Their Mediators in Reproduction, Pregnancy and Labour. Hum. Reprod. Update.

[50] Woidacki K., Jensen F., Zenclussen A.C. (2013). Mast Cells as Novel Mediators of Reproductive Processes. Front. Immunol..

[51] Füreder W., Bankl H.C., Toth J., Walchshofer S., Sperr W., Agis H., Semper H., Sillaber C., Lechner K., Valent P. (1997). Immunophenotypic and Functional Characterization of Human Tonsillar Mast Cells. J. Leukoc. Biol..

[52] Kadeh H., Derakhshanfar G., Saravani S. (2016). Comparative Study of Mast Cell Count in Oral Reactive Lesions and Its Association with Inflammation. Turk. J. Pathol..

[53] Mogoantă C.A., Ion D.A., Budu V., Muţiu G., Salplahta D., Afrem E. (2013). Evaluation of Microvascular Density in Inflammatory Lesions and Carcinoma of Palatine Tonsil. Rom. J. Morphol. Embryol..

[54] Brockmeyer P., Kling A., Schulz X., Perske C., Schliephake H., Hemmerlein B. (2017). High Mast Cell Density Indicates a Longer Overall Survival in Oral Squamous Cell Carcinoma. Sci. Rep..

[55] Ishikawa K., Yagi-Nakanishi S., Nakanishi Y., Kondo S., Tsuji A., Endo K., Wakisaka N., Murono S., Yoshizaki T. (2014). Expression of Interleukin-33 Is Correlated with Poor Prognosis of Patients with Squamous Cell Carcinoma of the Tongue. Auris Nasus Larynx.

[56] Molderings G.J., Afrin L.B. (2023). A Survey of the Currently Known Mast Cell Mediators with Potential Relevance for Therapy of Mast Cell-Induced Symptoms. Naunyn. Schmiedebergs. Arch. Pharmacol..

[57] Komi D.E.A., Redegeld F.A. (2020). Role of Mast Cells in Shaping the Tumor Microenvironment. Clin. Rev. Allergy Immunol..

[58] Shi S., Ye L., Yu X., Jin K., Wu W. (2023). Focus on Mast Cells in the Tumor Microenvironment: Current Knowledge and Future Directions. Biochim. Biophys. Acta—Rev. Cancer.

[59] Morita H., Arae K., Unno H., Miyauchi K., Toyama S., Nambu A., Oboki K., Ohno T., Motomura K., Matsuda A. (2015). An Interleukin-33-Mast Cell-Interleukin-2 Axis Suppresses Papain-Induced Allergic Inflammation by Promoting Regulatory T Cell Numbers. Immunity.

[60] Lv Y., Tian W., Teng Y., Wang P., Zhao Y., Li Z., Tang S., Chen W., Xie R., Lü M. (2024). Tumor-Infiltrating Mast Cells Stimulate ICOS+ Regulatory T Cells through an IL-33 and IL-2 Axis to Promote Gastric Cancer Progression. J. Adv. Res..

[61] Segura-Villalobos D., Ramírez-Moreno I.G., Martínez-Aguilar M., Ibarra-Sánchez A., Muñoz-Bello J.O., Anaya-Rubio I., Padilla A., Macías-Silva M., Lizano M., González-Espinosa C. (2022). Mast Cell–Tumor Interactions: Molecular Mechanisms of Recruitment, Intratumoral Communication and Potential Therapeutic Targets for Tumor Growth. Cells.

[62] Ménasché G., Longé C., Bratti M., Blank U. (2021). Cytoskeletal Transport, Reorganization, and Fusion Regulation in Mast Cell-Stimulus Secretion Coupling. Front. Cell Dev. Biol..

[63] Gangwar R.S., Landolina N., Arpinati L., Levi-Schaffer F. (2017). Mast Cell and Eosinophil Surface Receptors as Targets for Anti-Allergic Therapy. Pharmacol. Ther..

[64] Tauber M., Basso L., Martin J., Bostan L., Pinto M.M., Thierry G.R., Houmadi R., Serhan N., Loste A., Blériot C. (2023). Landscape of Mast Cell Populations across Organs in Mice and Humans. J. Exp. Med..

[65] St. John A.L., Abraham S.N. (2013). Innate Immunity and Its Regulation by Mast Cells. J. Immunol..

[66] Jiménez M., Cervantes-García D., Córdova-Dávalos L.E., Pérez-Rodríguez M.J., Gonzalez-Espinosa C., Salinas E. (2021). Responses of Mast Cells to Pathogens: Beneficial and Detrimental Roles. Front. Immunol..

[67] Blank U., Huang H., Kawakami T. (2021). The High Affinity IgE Receptor: A Signaling Update. Curr. Opin. Immunol..

[68] Gomez G., Gonzalez-Espinosa C., Odom S., Baez G., Cid M.E., Ryan J.J., Rivera J. (2005). Impaired FcεRI-Dependent Gene Expression and Defective Eicosanoid and Cytokine Production as a Consequence of Fyn Deficiency in Mast Cells. J. Immunol..

[69] Klemm S., Gutermuth J., Hültner L., Sparwasser T., Behrendt H., Peschel C., Mak T.W., Jakob T., Ruland J. (2006). The Bcl10–Malt1 Complex Segregates FcεRI-Mediated Nuclear Factor ΚB Activation and Cytokine Production from Mast Cell Degranulation. J. Exp. Med..

[70] Sandig H., Bulfone-Paus S. (2012). TLR Signaling in Mast Cells: Common and Unique Features. Front. Immunol..

[71] Takeuchi O., Akira S. (2010). Pattern Recognition Receptors and Inflammation. Cell.

[72] Sundaram B., Tweedell R.E., Prasanth Kumar S., Kanneganti T.-D. (2024). The NLR Family of Innate Immune and Cell Death Sensors. Immunity.

[73] Yoneyama M., Kato H., Fujita T. (2024). Physiological Functions of RIG-I-like Receptors. Immunity.

[74] Cheng S., Li Z., Gao R., Xing B., Gao Y., Yang Y., Qin S., Zhang L., Ouyang H., Du P. (2021). A Pan-Cancer Single-Cell Transcriptional Atlas of Tumor Infiltrating Myeloid Cells. Cell.

[75] Guo X., Sun M., Yang P., Meng X., Liu R. (2023). Role of Mast Cells Activation in the Tumor Immune Microenvironment and Immunotherapy of Cancers. Eur. J. Pharmacol..

[76] Zhang L., Pan J., Wang Z., Yang C., Chen W., Jiang J., Zheng Z., Jia F., Zhang Y., Jiang J. (2022). Multi-Omics Profiling Suggesting Intratumoral Mast Cells as Predictive Index of Breast Cancer Lung Metastasis. Front. Oncol..

[77] Mao Y., Feng Q., Zheng P., Yang L., Zhu D., Chang W., Ji M., He G., Xu J. (2018). Low Tumor Infiltrating Mast Cell Density Confers Prognostic Benefit and Reflects Immunoactivation in Colorectal Cancer. Int. J. Cancer.

[78] Yin H., Wang X., Jin N., Ling X., Leng X., Wang Y., Ma K., Jiang X., Zhu J., Ma J. (2021). Integrated Analysis of Immune Infiltration in Esophageal Carcinoma as Prognostic Biomarkers. Ann. Transl. Med..

[79] Liu Z., Zhu Y., Xu L., Zhang J., Xie H., Fu H., Zhou Q., Chang Y., Dai B., Xu J. (2018). Tumor Stroma-Infiltrating Mast Cells Predict Prognosis and Adjuvant Chemotherapeutic Benefits in Patients with Muscle Invasive Bladder Cancer. Oncoimmunology.

[80] Hempel H.A., Cuka N.S., Kulac I., Barber J.R., Cornish T.C., Platz E.A., De Marzo A.M., Sfanos K.S. (2017). Low Intratumoral Mast Cells Are Associated With a Higher Risk of Prostate Cancer Recurrence. Prostate.

[81] Hempel Sullivan H., Heaphy C.M., Kulac I., Cuka N., Lu J., Barber J.R., De Marzo A.M., Lotan T.L., Joshu C.E., Sfanos K.S. (2020). High Extratumoral Mast Cell Counts Are Associated with a Higher Risk of Adverse Prostate Cancer Outcomes. Cancer Epidemiol. Biomark. Prev..

[82] Johansson A., Rudolfsson S., Hammarsten P., Halin S., Pietras K., Jones J., Stattin P., Egevad L., Granfors T., Wikström P. (2010). Mast Cells Are Novel Independent Prognostic Markers in Prostate Cancer and Represent a Target for Therapy. Am. J. Pathol..

[83] Ramírez-Moreno I.G., Ibarra-Sánchez A., Castillo-Arellano J.I., Blank U., González-Espinosa C. (2020). Mast Cells Localize in Hypoxic Zones of Tumors and Secrete CCL-2 under Hypoxia through Activation of L-Type Calcium Channels. J. Immunol..

[84] Maltby S., Khazaie K., McNagny K.M. (2009). Mast Cells in Tumor Growth: Angiogenesis, Tissue Remodelling and Immune-Modulation. Biochim. Biophys. Acta—Rev. Cancer.

[85] Lichterman J.N., Reddy S.M. (2021). Mast Cells: A New Frontier for Cancer Immunotherapy. Cells.

[86] Solimando A.G., Desantis V., Ribatti D. (2022). Mast Cells and Interleukins. Int. J. Mol. Sci..

[87] Derakhshani A., Vahidian F., Alihasanzadeh M., Mokhtarzadeh A., Lotfi Nezhad P., Baradaran B. (2019). Mast Cells: A Double-Edged Sword in Cancer. Immunol. Lett..

[88] Kalyani R., Rajeshwari G. (2016). Significance of Mast Cells in Non-Neoplastic and Neoplastic Lesions of Uterine Cervix. Biomed. Res. Ther..

[89] Naik R., Pai M.R., Poornima Baliga B., Nayak K.S., Shankarnarayana, Dighe P. (2004). Mast Cell Profile in Uterine Cervix. Indian J. Pathol. Microbiol..

[90] Punt S., Fleuren G.J., Kritikou E., Lubberts E., Trimbos J.B., Jordanova E.S., Gorter A. (2015). Angels and Demons: Th17 Cells Represent a Beneficial Response, While Neutrophil IL-17 Is Associated with Poor Prognosis in Squamous Cervical Cancer. Oncoimmunology.

[91] Benítez-Bribiesca L., Wong A., Utrera D., Castellanos E. (2001). The Role of Mast Cell Tryptase in Neoangiogenesis of Premalignant and Malignant Lesions of the Uterine Cervix. J. Histochem. Cytochem..

[92] Utrera-Barillas D., Castro-Manrreza M., Castellanos E., Gutiérrez-Rodríguez M., Arciniega-Ruíz de Esparza O., García-Cebada J., Velazquez J.R., Flores-Reséndiz D., Hernández-Hernández D., Benítez-Bribiesca L. (2010). The Role of Macrophages and Mast Cells in Lymphangiogenesis and Angiogenesis in Cervical Carcinogenesis. Exp. Mol. Pathol..

[93] Mondal S., Dasgupta S., Mandal P., Chatterjee S., Chakraborty D. (2014). Is There Any Role of Mast Cell Density and Microvessel Density in Cervical Squamous Cell Carcinoma? A Histologic Study with Special Reference to CD-34 Immunomarker Staining. Indian J. Med. Paediatr. Oncol..

[94] Cabanillas-Saez A., Schalper J.A., Nicovani S.M., Rudolph M.I. (2002). Characterization of Mast Cells According to Their Content of Tryptase and Chymase in Normal and Neoplastic Human Uterine Cervix. Int. J. Gynecol. Cancer.

[95] Diaconu N.-C., Rummukainen J., Naukkarinen A., Mättö M., Harvima R.J., Pelkonen J., Harvima I.T. (2011). Mast Cell Chymase Is Present in Uterine Cervical Carcinoma and It Detaches Viable and Growing Cervical Squamous Carcinoma Cells from Substratum in Vitro. Arch. Dermatol. Res..

[96] Wang J., Li Z., Gao A., Wen Q., Sun Y. (2019). The Prognostic Landscape of Tumor-Infiltrating Immune Cells in Cervical Cancer. Biomed. Pharmacother..

[97] Wu Z., Lin Q., Sheng L., Chen W., Liang M., Wu D., Ke Y. (2023). A Novel Immune-Related Risk-Scoring System Associated with the Prognosis and Response of Cervical Cancer Patients Treated with Radiation Therapy. Front. Mol. Biosci..

[98] Huang B., Zheng J., Chen B., Wu M., Xiao L. (2025). Analysis of the Correlation between RFC4 Expression and Tumor Immune Microenvironment and Prognosis in Patients with Cervical Cancer. Front. Genet..

[99] Wang L., Liu H., Feng Y., Liu X., Wang Y., Liu Y., Li H., Zhang Y. (2024). Decoding the Immune Landscape: A Comprehensive Analysis of Immune-Associated Biomarkers in Cervical Carcinoma and Their Implications for Immunotherapy Strategies. Front. Genet..

[100] Mo X., Wang N., He Z., Kang W., Wang L., Han X., Yang L. (2023). The Sub-Molecular Characterization Identification for Cervical Cancer. Heliyon.

[101] Zhao F., Hong J., Zhou G., Huang T., Lin Z., Zhang Y., Liang L., Tang H. (2024). Elucidating the Role of Tumor-Associated ALOX5+ Mast Cells with Transformative Function in Cervical Cancer Progression via Single-Cell RNA Sequencing. Front. Immunol..

[102] Xue L., Gao L., Zhou S., Yan C., Zhang X., Lin W., Li H., Shen Y., Wang X. (2025). Single-Cell RNA Sequencing Revealed Changes in the Tumor Microenvironment Induced by Radiotherapy for Cervical Cancer and the Molecular Mechanism of Mast Cells in Immunosuppression. Funct. Integr. Genom..

[103] Rudolph M.I., Boza Y., Yefi R., Luza S., Andrews E., Penissi A., Garrido P., Rojas I.G. (2008). The Influence of Mast Cell Mediators on Migration of SW756 Cervical Carcinoma Cells. J. Pharmacol. Sci..

[104] Muñoz-Bello J.O., Romero-Córdoba S.L., García-Chávez J.N., González-Espinosa C., Langley E., Lizano M. (2024). Potential Transcript-Based Biomarkers Predicting Clinical Outcomes of HPV-Positive Head and Neck Squamous Cell Carcinoma Patients. Cells.

[105] Johnson D.E., Burtness B., Leemans C.R., Lui V.W.Y., Bauman J.E., Grandis J.R. (2020). Head and Neck Squamous Cell Carcinoma. Nat. Rev. Dis. Prim..

[106] Laishram D., Rao K., Devi H.S.U., Priya N.S., Smitha T., Sheethal H.S. (2017). Mast Cells and Angiogenesis in Malignant and Premalignant Oral Lesions: An Immunohistochemical Study. J. Oral Maxillofac. Pathol..

[107] Kurihara-Shimomura M., Sasahira T., Shimomura H., Bosserhoff Katrin A., Kirita T. (2020). Mast Cell Chymase Promotes Angiogenesis and Lymphangiogenesis Mediated by Activation of Melanoma Inhibitory Activity Gene Family Members in Oral Squamous Cell Carcinoma. Int. J. Oncol..

[108] Tzorakoleftheraki S.-E., Koletsa T. (2024). The Complex Role of Mast Cells in Head and Neck Squamous Cell Carcinoma: A Systematic Review. Medicina.

[109] Newman A.M., Liu C.L., Green M.R., Gentles A.J., Feng W., Xu Y., Hoang C.D., Diehn M., Alizadeh A.A. (2015). Robust Enumeration of Cell Subsets from Tissue Expression Profiles. Nat. Methods.

[110] Cai Z., Tang B., Chen L., Lei W. (2022). Mast Cell Marker Gene Signature in Head and Neck Squamous Cell Carcinoma. BMC Cancer.

[111] Ding Y., Chu L., Cao Q., Lei H., Li X., Zhuang Q. (2023). A Meta-Validated Immune Infiltration-Related Gene Model Predicts Prognosis and Immunotherapy Sensitivity in HNSCC. BMC Cancer.

[112] Lin Y., Wu F., Huang X., Zhang Z., Liu C., Lin Y., Xu Y., Guo H., Hong C. (2025). A Novel Mast Cell Marker Gene-Related Prognostic Signature to Predict Prognosis and Reveal the Immune Landscape in Head and Neck Squamous Cell Carcinoma. Front. Immunol..

[113] Chen F., Gong X., Xia M., Yu F., Wu J., Yu C., Li J. (2022). The Aging-Related Prognostic Signature Reveals the Landscape of the Tumor Immune Microenvironment in Head and Neck Squamous Cell Carcinoma. Front. Oncol..

[114] Attramadal C.G., Kumar S., Gao J., Boysen M.E., Halstensen T.S., Bryne M. (2016). Low Mast Cell Density Predicts Poor Prognosis in Oral Squamous Cell Carcinoma and Reduces Survival in Head and Neck Squamous Cell Carcinoma. Anticancer Res..

[115] Zhang L., Wang W.-Q., Chen J.-H., Feng J., Liao Y.-Z., Zou Y., Liu R. (2024). Tumor-Infiltrating Immune Cells and Survival in Head and Neck Squamous Cell Carcinoma: A Retrospective Computational Study. Sci. Rep..

[116] Zhou D., Wang J., Wang J., Liu X. (2021). Profiles of Immune Cell Infiltration and Immune-Related Genes in the Tumor Microenvironment of HNSCC with or without HPV Infection. Am. J. Transl. Res..

[117] Tosi A., Parisatto B., Menegaldo A., Spinato G., Guido M., Del Mistro A., Bussani R., Zanconati F., Tofanelli M., Tirelli G. (2022). The Immune Microenvironment of HPV-Positive and HPV-Negative Oropharyngeal Squamous Cell Carcinoma: A Multiparametric Quantitative and Spatial Analysis Unveils a Rationale to Target Treatment-Naïve Tumors with Immune Checkpoint Inhibitors. J. Exp. Clin. Cancer Res..

[118] Wang Z., Wang Q., Tao Y., Chen J., Yuan Z., Wang P. (2023). Characterization of Immune Microenvironment in Patients with HPV-Positive and Negative Head and Neck Cancer. Sci. Data.

[119] Coussens L.M., Hanahan D., Arbeit J.M. (1996). Genetic Predisposition and Parameters of Malignant Progression in K14-HPV16 Transgenic Mice. Am. J. Pathol..

[120] Coussens L.M., Raymond W.W., Bergers G., Laig-Webster M., Behrendtsen O., Werb Z., Caughey G.H., Hanahan D. (1999). Inflammatory Mast Cells Up-Regulate Angiogenesis during Squamous Epithelial Carcinogenesis. Genes Dev..

[121] Bergot A.-S., Ford N., Leggatt G.R., Wells J.W., Frazer I.H., Grimbaldeston M.A. (2014). HPV16-E7 Expression in Squamous Epithelium Creates a Local Immune Suppressive Environment via CCL2- and CCL5- Mediated Recruitment of Mast Cells. PLoS Pathog..

[122] Ghouse S.M., Polikarpova A., Muhandes L., Dudeck J., Tantcheva-Poór I., Hartmann K., Lesche M., Dahl A., Eming S., Müller W. (2018). Although Abundant in Tumor Tissue, Mast Cells Have No Effect on Immunological Micro-Milieu or Growth of HPV-Induced or Transplanted Tumors. Cell Rep..

[123] Costa A.C., Santos J.M.O., Medeiros-Fonseca B., Oliveira P.A., Bastos M.M.S.M., Brito H.O., Gil da Costa R.M., Medeiros R. (2022). Characterizing the Inflammatory Microenvironment in K14-HPV16 Transgenic Mice: Mast Cell Infiltration and MicroRNA Expression. Cancers.

[124] Lyons J.J., Metcalfe D.D. (2020). Targeting Mast Cells with Biologics. Immunol. Allergy Clin. N. Am..

[125] Ribatti D. (2024). New Insights into the Role of Mast Cells as a Therapeutic Target in Cancer through the Blockade of Immune Checkpoint Inhibitors. Front. Med..

[126] Siebenhaar F., Metz M., Maurer M. (2014). Mast Cells Protect from Skin Tumor Development and Limit Tumor Growth during Cutaneous de Novo Carcinogenesis in a Kit-Dependent Mouse Model. Exp. Dermatol..

[127] Bodduluri S.R., Mathis S., Maturu P., Krishnan E., Satpathy S.R., Chilton P.M., Mitchell T.C., Lira S., Locati M., Mantovani A. (2018). Mast Cell–Dependent CD8+ T-Cell Recruitment Mediates Immune Surveillance of Intestinal Tumors in ApcMin/+ Mice. Cancer Immunol. Res..

[128] Shibata T., Lieblong B.J., Sasagawa T., Nakagawa M. (2019). The Promise of Combining Cancer Vaccine and Checkpoint Blockade for Treating HPV-Related Cancer. Cancer Treat. Rev..

[129] Han X., Chang W., Xia X. (2022). Immune Checkpoint Inhibitors in Advanced and Recurrent/Metastatic Cervical Cancer. Front. Oncol..

[130] Cao K., Zhang G., Zhang X., Yang M., Wang Y., He M., Lu J., Liu H. (2021). Stromal Infiltrating Mast Cells Identify Immunoevasive Subtype High-Grade Serous Ovarian Cancer with Poor Prognosis and Inferior Immunotherapeutic Response. Oncoimmunology.

[131] Somasundaram R., Connelly T., Choi R., Choi H., Samarkina A., Li L., Gregorio E., Chen Y., Thakur R., Abdel-Mohsen M. (2021). Tumor-Infiltrating Mast Cells Are Associated with Resistance to Anti-PD-1 Therapy. Nat. Commun..

[132] Li J., Peng G., Zhu K., Jie X., Xu Y., Rao X., Xu Y., Chen Y., Xing B., Wu G. (2023). PD-1+ Mast Cell Enhanced by PD-1 Blocking Therapy Associated with Resistance to Immunotherapy. Cancer Immunol. Immunother..

[133] Panagi M., Mpekris F., Voutouri C., Hadjigeorgiou A.G., Symeonidou C., Porfyriou E., Michael C., Stylianou A., Martin J.D., Cabral H. (2024). Stabilizing Tumor-Resident Mast Cells Restores T-Cell Infiltration and Sensitizes Sarcomas to PD-L1 Inhibition. Clin. Cancer Res..

[134] Lv Y., Zhao Y., Wang X., Chen N., Mao F., Teng Y., Wang T., Peng L., Zhang J., Cheng P. (2019). Increased Intratumoral Mast Cells Foster Immune Suppression and Gastric Cancer Progression through TNF-α-PD-L1 Pathway. J. Immunother. Cancer.

[135] Soucek L., Lawlor E.R., Soto D., Shchors K., Swigart L.B., Evan G.I. (2007). Mast Cells Are Required for Angiogenesis and Macroscopic Expansion of Myc-Induced Pancreatic Islet Tumors. Nat. Med..

[136] Melillo R.M., Guarino V., Avilla E., Galdiero M.R., Liotti F., Prevete N., Rossi F.W., Basolo F., Ugolini C., de Paulis A. (2010). Mast Cells Have a Protumorigenic Role in Human Thyroid Cancer. Oncogene.

[137] Eissmann M.F., Dijkstra C., Jarnicki A., Phesse T., Brunnberg J., Poh A.R., Etemadi N., Tsantikos E., Thiem S., Huntington N.D. (2019). IL-33-Mediated Mast Cell Activation Promotes Gastric Cancer through Macrophage Mobilization. Nat. Commun..

[138] Aliabadi A., Haghshenas M.R., Kiani R., Panjehshahin M.R., Erfani N. (2024). Promising Anticancer Activity of Cromolyn in Colon Cancer: In Vitro and in Vivo Analysis. J. Cancer Res. Clin. Oncol..

[139] Jeong H.J., Oh H.A., Nam S.Y., Han N.R., Kim Y.S., Kim J.H., Lee S.J., Kim M.H., Moon P.D., Kim H.M. (2013). The Critical Role of Mast Cell-Derived Hypoxia-Inducible Factor-1α in Human and Mice Melanoma Growth. Int. J. Cancer.

[140] Chen S., Luster A.D. (2022). Antihistamines for Cancer Immunotherapy: More than Just Treating Allergies. Cancer Cell.

[141] Ammendola M., Leporini C., Marech I., Gadaleta C.D., Scognamillo G., Sacco R., Sammarco G., De Sarro G., Russo E., Ranieri G. (2014). Targeting Mast Cells Tryptase in Tumor Microenvironment: A Potential Antiangiogenetic Strategy. BioMed Res. Int..

[142] Oldford S.A., Haidl I.D., Howatt M.A., Leiva C.A., Johnston B., Marshall J.S. (2010). A Critical Role for Mast Cells and Mast Cell-Derived IL-6 in TLR2-Mediated Inhibition of Tumor Growth. J. Immunol..

[143] Drobits B., Holcmann M., Amberg N., Swiecki M., Grundtner R., Hammer M., Colonna M., Sibilia M. (2012). Imiquimod Clears Tumors in Mice Independent of Adaptive Immunity by Converting PDCs into Tumor-Killing Effector Cells. J. Clin. Investig..

[144] Lyng H., Malinen E. (2017). Hypoxia in Cervical Cancer: From Biology to Imaging. Clin. Transl. Imaging.

[145] Li J.Z., Gao W., Chan J.Y.-W., Ho W.-K., Wong T.-S. (2012). Hypoxia in Head and Neck Squamous Cell Carcinoma. Int. Sch. Res. Not..

[146] Ruiz F.J., Inkman M., Rashmi R., Muhammad N., Gabriel N., Miller C.A., McLellan M.D., Goldstein M., Markovina S., Grigsby P.W. (2021). HPV Transcript Expression Affects Cervical Cancer Response to Chemoradiation. JCI Insight.

